# Prevalent hepatitis B surface antigen among first-time blood donors in Gabon

**DOI:** 10.1371/journal.pone.0194285

**Published:** 2018-04-13

**Authors:** Jean Marie Eko Mba, Cyrille Bisseye, Jophrette Mireille Ntsame Ndong, Landry Erik Mombo, Calixte Bengone, Guy Mouelet Migolet, Bertrand M’batchi, Heidi E. Kosiorek, Richard J. Butterfield, Lewis R. Roberts, Mitesh J. Borad, Bolni Marius Nagalo

**Affiliations:** 1 Centre National de Transfusion sanguine, Libreville, Gabon; 2 Laboratoire de Biologie Moléculaire et Cellulaire (LABMC), Université des Sciences et Techniques de Masuku, Franceville, Gabon; 3 Division of Biostatistics, Mayo Clinic, Scottsdale, Arizona, United States of America; 4 Division of Gastroenterology and Hepatology, Mayo Clinic College of Medicine, Rochester, Minnesota, United States of America; 5 Division of Hematology/Oncology, Mayo Clinic, Scottsdale, Arizona, United States of America; FDA, UNITED STATES

## Abstract

Despite chronic Hepatitis B virus (HBV) infection being the main cause of younger-onset complex liver disease including cirrhosis and hepatocellular carcinoma (HCC) in Africa, very little is known regarding the seroprevalence of HBV in the Gabonese general population. This investigation aimed to provide strong epidemiological data and risk factors associated with HBV infection in first-time blood donors representative of the urban adult population. The screening of HBsAg was carried out using 4th generation ELISA kits. The overall seroprevalence of HBsAg was 7.28%. The frequency of HBsAg was differential and marked by annual variations in blood donors from 2009 to 2016. Seroprevalence was 2-fold higher among males versus females (OR = 1.90 (95% CI: 1.75–2.06), P<0.001). HBsAg seroprevalence was significantly higher in donors of the age group 25–35 years old compared to donors of the age group <18 years (OR = 1.64 (95% CI: 1.03–2.60), P = 0.04). The seroprevalence of HBsAg in family/replacement donors (FRD) was significantly higher than that of voluntary non-remunerated donors (VNRD) (OR = 0.88 (95% CI: 0.83–0.94), P <0.001). The simultaneous comparison of HBsAg seroprevalence with blood donation type, gender and age showed that the higher prevalence in FRD was significant only in males between 18 and 45 years and in females between 25 and 34 years of age. This study confirms the high endemicity of HBV in Gabon while identifying the most infected age groups for both men and women.

## Introduction

The hepatitis B virus is responsible for a highly contagious infectious disease that is transmitted through sexual intercourse or from mother to child during pregnancy, delivery or breastfeeding, blood transfusion. In spite of the availability of an effective vaccine against hepatitis B virus (HBV), this infection remains a major public health concern to date. Chronic HBV is directly or indirectly responsible for a high number of human deaths regardless of the age, gender or socioeconomic status in Sub-Saharan Africa.

In 2015, the World Health Organization (WHO) reported an estimated 325 million people worldwide living with chronic HBV, in which 60 million people were Africans [1].

In addition, liver cancers induced by chronic HBV infection cause the death of nearly 600,000 individuals each year [2]. Sub-Saharan Africans are developing liver cancer at much younger age and faster rate compared to Asians and Caucasians [3].

People with HBV are at risk of developing chronicity with the onset of complications. Similarly, 90% of infants born from mothers carrying hepatitis B surface antigen (HBsAg) and infants infected in the first year of life will most likely develop chronic infection. Contrastingly, among adolescents and adults the majority of whom are infected via sexual intercourse, exposure to contaminated blood products or materials (syringes and needles), 2 to 5% will turn into chronic carriers [2, 4].

In sub-Saharan Africa, 12.5% of transfused patients are at a risk of posttransfusional hepatitis despite transfusion safety measures including rigorous donor selection and biological qualification of blood donation through adequate virological testing [5, 6].

Previous studies in Gabon have reported varying seroprevalence of HBV. Two studies in the 1990s reported high seroprevalence of 9.2% and 19% [7, 8]. Variable seroprevalence of HBV has been reported in various populations such as 9.2% in pregnant women; 12.9% and 16.7% in urban and rural populations respectively; and 5.63% in blood donors [9–12].

However, according to WHO, Gabon is clustered in countries with high HBV endemicity [13]. There is a serious need to assess the real prevalence of HBV infection in the Gabonese population in order to develop appropriate approaches to reduce the burden of this disease. However, a limitation in developing such a strategy is the shortage of strong data on HBV seroprevalence in the general population. While the prevalence of hepatitis B was reported for the first time in 1988 and 1989 [12, 14], vaccination was introduced in 2004 with the Expanded Program on Immunization (EPI). However, its impact on the HBsAg seroprevalence in the general population remains unknown. We describe an 8 year study compiling HBV seroprevalence and risk factors associated with its transmission in a population of first-time blood donors representative of the adult urban population.

## Materials and methods

### Study site

The study was carried out at the National Center for Blood Transfusion (NBTC), which oversees all components of the blood donation chain from collection, screening for transfusion transmitted diseases (TTIs) and distribution of labile blood products. The center is located in the Gabonese capital, Libreville, with a cosmopolitan population of 703,940 inhabitants [15], comprising just over one third of the Gabonese population estimated at 1,811,079 inhabitants.

### Blood donors

A retrospective analysis of blood donor data from 2009 to 2016 was carried out at the NBTC. All apparently healthy voluntary non-remunerated donors (VNRD) and family / replacement donors (FRD) were selected after informed consent and completion of a panel of questions including their medical history. Individuals aged 15 to 65 years old with a weight ≥ 50 kg were eligible for blood donations. Candidates with multi-transfusion history, individuals with jaundice or signs of hepatitis, pregnant women and persons who displayed unsafe sexual behavior during the six months prior to donation of blood were excluded. Venous blood was collected in the blood packs following standard procedures.

### Serological assays

All blood donors were tested for the hepatitis B surface antigen with a commercially available enzyme immunoassay. All samples reactive to the first test were retested with a second ELISA. Only samples reactive to both tests were considered positive. The tests used HBsAg from 2009 to 2016 are shown in Table 1.

**Table 1:** Immunoassays used in the screening of HBsAg in blood donors from 2009 to 2016.

Years	Assays	Specificity	Sensitivity	Manufacturer	Origin
2009	Monolisa HBs Ag-Ab ULTRA	100%	100%	Bio-Rad	France
	Hepanostika HBsAg	99.94%	100%	Biomérieux	France
2010	Monolisa HBs Ag-Ab ULTRA	100%	100%	Bio-Rad	France
	Hepanostika HBsAg	99.94%	100%	Biomérieux	France
2011	Monolisa HBs Ag-Ab ULTRA	100%	100%	Bio-Rad	France
	Hepanostika HBsAg	99.94%	100%	Biomérieux	France
2012	Monolisa HBs Ag-Ab ULTRA	100%	100%	Bio-Rad	France
	Hepanostika HBsAg	99.94%	100%	Biomérieux	France
2013	Monolisa HBs Ag-Ab ULTRA	100%	100%	Bio-Rad	France
	Vikia AgHBs	99.80%	99.05%	Biomérieux	France
	Hep Check	100%	97.26%	Veda Lab	France
	ARCHITECT	99.87%	99.52%	Abbott	USA
2014	Monolisa HBs Ag-Ab ULTRA	100%	100%	Bio-Rad	France
	ARCHITECT	99.87%	99.52%	Abbott	USA
	Vidas HBs Ultra	100%	100%	Biomérieux	France
2015	Monolisa HBs Ag-Ab ULTRA	100%	100%	Bio-Rad	France
	ARCHITECT	99.87%	99.52%	Abbott	USA
	AgHBs Determine	96.6%	100%	Alere	France
2016	Monolisa HBs Ag-Ab ULTRA	100%	100%	Bio-Rad	France
	Cobas 600 e601	100%	100%	Roche	France

Sensitivity and specificity presented are from the manufacturers

### Ethical considerations

This study was approved by the NBTC ethics committee. All study participants and parents or guardians of blood donors <18 years old gave their free written and informed consent.

### Statistical analysis

HBsAg seroprevalence was described by year and according to demographic characteristics (gender, age group, occupation and donor type.). Logistic regression was used to assess the impact of these characteristics on seroprevalence in both univariate and multivariable analysis. Odds ratios (OR) and 95% confidence intervals are presented. SAS version 9.4 (Cary, NC) was used for analysis. P values < 0.05 were considered statistically significant.

## Results

### Sociodemographic characteristics of blood donors

A total of 69,862 first-time blood donors were recruited from 2009 to 2016. Blood donors were predominantly male (76.4%). The proportion of male blood donors has fluctuated from 71.0% in 2011 to 85.3% in 2015 (Table 2). The average age of blood donors was 28.5 ± 7.8 years with a minimum age of 15 years and a maximum age of 65 years. Overall, among blood donors the largest group age was 25–35 years (43.9%). Blood donors ≤35 years old accounted for almost 82% of blood donors. However, the age group 18–24 years was the most represented in blood donors in 2010 with 43.9% (Table 2). Family/replacement donors (FRD) and voluntary non-remunerated donors (VNRD) accounted for 63.4% and 36.6% of all blood donors. VNRD were more represented than FRD in 2010 with 54.8% (Table 2). Concerning the socio-professional categories, students accounted for 27.4% of blood donors followed by police and armed forces (17.7%). The "other occupations" group, comprising several socio-professional categories, accounted for 28.2% of first-time blood donors. The least represented occupations were hotel and restaurant workers (1.1%), healthcare workers (1.4%) and teachers-researchers (2.9%) (Table 2).

**Table 2:** Sociodemographic characteristics of blood donors from 2009 to 2016.

	2009	2010	2011	2012	2013	2014	2015	2016	Total
**Number of donors**	7331	8327	9812	9441	10427	10428	4430	9666	69862
**Gender N (%)**									
Female	1346 (18.36)	2299 (27.61)	2847 (29.02)	1767 (18.72)	2969 (28.47)	2363 (22.66)	652 (14.72)	2229 (23.06)	16472 (23.58)
Male	5985 (81.64)	6028 (72.39	6965 (70.98)	7674 (81.28)	7458 (71.53)	8065 (77.34)	3778 (85.28)	7437 (76.94)	53390 (76.42)
**Age groups N (%)**									
<18 years	20 (0.27)	88 (1.06)	72 (0.73)	19 (0.20)	94 (0.90)	75 (0.72)	2 (0.05)	43 (0.44)	413 (0.59)
18–24 years	2249 (30.68)	3655 (43.89)	3830 (39.03)	3659 (38.76)	4046 (38.80)	4003 (38.39)	1333 (30.09)	3167 (32.76)	25942 (37.13)
25–35 years	3562 (48.59)	3113 (37.38)	3949 (40.25)	4117 (43.61)	4381 (42.02)	4507 (43.22)	2250 (50.79)	4770 (49.35)	30649 (43.87)
36–45 years	1197 (16.33)	1089 (13.08)	1483 (15.11)	1279 (13.55)	1515 (14.53)	1467 (14.07)	674 (15.21)	1340 (13.86)	10044 (14.38)
> 45 years	303 (4.13)	382 (4.59)	478 (4.87)	367 (3.89)	391 (3.75)	376 (3.61)	171 (3.86)	346 (3.58)	2814 (4.03)
**Blood donors N (%)**									
VNRD	3072 (41.90)	4565 (54.82)	4296 (43.78)	4144 (43.89)	4261 (40.87)	2856 (27.39)	636 (14.36)	1764 (18.25)	25594 (36.64)
FRD	4259 (58.10)	3762 (45.18)	5516 (56.22)	5297 (56.11)	6166 (59.13)	7572 (72.61)	3794 (85.64)	7902 (81.75)	44268 (63.36)
**Occupation N (%)**									
Healthcare workers	186 (2.54)	100 (1.20)	143 (1.46)	146 (1.55)	124 (1.19)	116 (1.11)	75 (1.69)	91 (0.94)	981 (1.40)
Students	1979 (26.99)	1538 (18.47)	2227 (22.70)	2861 (30.30	3061 (29.36)	3260 (31.26)	1672 (31.74)	2539 (26.27)	19137 (27.39)
Teachers/Researchers	356 (4.86)	200 (2.40)	289 (2.95)	329 (3.48)	250 (2.40)	222 (2.13)	153 (3.45)	197 (2.04)	1996 (2.86)
Police and armed forces	1392 (18.99)	2495 (29.96)	1465 (14.93)	2194 (23.24)	1600 15.34)	1806 (17.32)	238 (5.37)	1172 (12.12)	12362 (17.69)
Drivers	472 (6.44)	390 (4.68)	496 (5.06)	495 (5.24)	546 (5.24)	552 (5.29)	323 (7.29)	479 (4.96)	3753 (5.37)
Goverment employees	408 (5.57)	266 (3.19)	253 (2.58)	384 (4.07)	309 (2.96)	352 (3.38)	174 (3.93)	280 (2.90)	2426 (3.47)
Tailors and Hairdressers	722 (9.85)	585 (7.03)	781 (7.96)	984 (10.42)	879 (8.43)	839 (8.05)	455 (10.27)	587 (6.07)	5832 (8.35)
Hotel and restaurant workers	96 (1.31)	78 (0.94)	97 (0.99)	99 (1.05)	104 (1.00)	107 (1.03)	71 (1.60)	88 (0.91)	740 (1.06)
Merchants	393 (5.36)	285 (3.42)	361 (3.68)	464 (4.91)	446 (4.28)	450 (4.32)	238 (5.37)	297 (3.07)	2934 (4.20)
Others	1327 (18.10)	2390 (28.70)	3700 (37.71)	1485 (15.73)	3108 (29.81)	2724 (26.12)	1031 (23.27)	3936 (40.72)	19701 (28.20)

VNRD = Voluntary non-remunerated donors; FRD = Family/replacement donors

### Overall seroprevalence of HBsAg in blood donors

**Fig 1:**
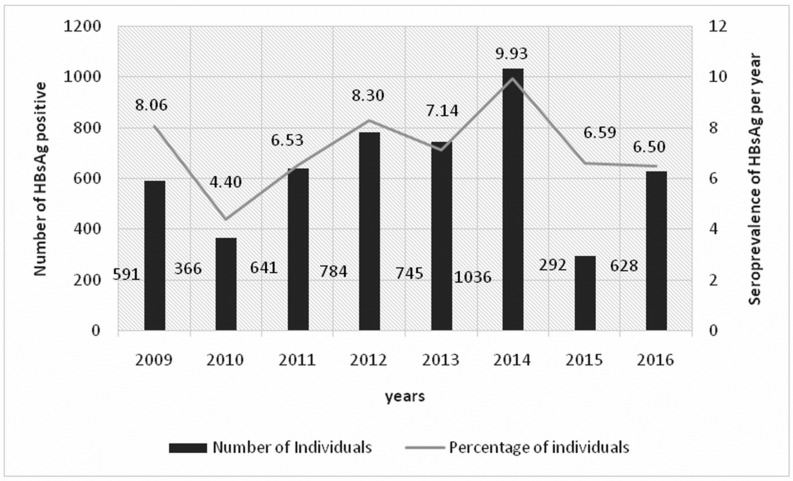
Cases and HBsAg frequency per year.

Of the 69,862 first-time donors, the overall seroprevalence of HBsAg was 7.28%. This seroprevalence experienced annual variation from 2009 to 2016. The lowest seroprevalence was observed in 2010 with 4.40% while the highest was reported in 2014 with 9.93%. The number of HBsAg positive blood donors increased steadily between 2010 and 2014 with 366/8327 and 1036/10428 cases, respectively, before decreasing significantly to 628/9666 in 2016 (Fig 1).

### Overall HBsAg seroprevalence according to sociodemographic characteristics of blood donors

On univariate analysis higher HBsAg seroprevalence was observed in male blood donors (OR = 1.90 (95% CI: 1.75–2.06), P< 0.001), the age groups 25–35 years (OR = 1.83 (95% CI: 1.15–2.90), P = 0.01) and 36–45 years (OR = 1.82 (95% CI: 1.14–2.90), P = 0.01), family/ replacement donors (OR = 1.33 (95% CI: 1.25–1.41), P< 0.001), students (OR = 1.17 (1.07–1.28), P = 0.001), drivers (OR = 1.47 (95% CI: 1.28–1.69), P< 0.001), tailors and hairdressers (OR = 1.53 (1.36–1.72), P< 0.001), hotels and restaurant workers (OR = 1.59 (1.22–2.06), P <0.001) and merchants (OR = 1.44 (95% CI: 1.24–1.67), P< 0.001) (Table 3). Similar results were seen based on multivariable logistic regression as male blood donors (OR = 1.90 (1.75–2.06), P< 0.001), the age group 25–35 years (OR = 1.64 (95% CI: 1.03–2.60), P = 0.04), family/ replacement donors (OR = 0.88 (95% CI: 0.83–0.94), P< 0.001), students (OR = 1.33 (1.21–1.46), P = 0.001), drivers (OR = 1.28 (95% CI: 1.11–1.47), P <0.001), tailors and hairdressers (OR = 1.47 (95% CI: 1.30–1.66), P< 0.001), hotels and restaurant workers (OR = 1.56 (1.20–2.03), P <0.001) and merchants (OR = 1.40 (1.20–1.63), P< 0.001) were shown to be associated with HBsAg seroprevalence (Table 3)

**Table 3:** Seroprevalence of HBsAg in all blood donors according to their socio-demographic characteristics from 2009 to 2016.

Variables	N	HBsAg (+)	Univariate		Multivariable	
			COR (95% CI)	^a^P-value	AOR (95% CI)	^b^P-value
**Gender**						
Female	16472	735 (4.46)	**Referent**			
Male	53390	4348 (8.14)	1.90 (1.75–2.06)	**<.001**	1.90 (1.75–2.06)	**<.001**
**Age groups**						
<18 years	413	19 (4.60)	**Referent**			
18–24 years	25942	1617 (6.23)	1.38 (0.87–2.19)	0.17	1.29 (0.81–2.05)	0.29
25–35 years	30649	2481 (8.09)	1.83 (1.15–2.90)	**0.01**	1.64 (1.03–2.60)	**0.04**
36–45 years	10044	811 (8.07)	1.82 (1.14–2.90)	**0.012**	1.59 (0.99–2.54)	0.05
> 45 years	2814	155 (5.51)	1.219 (0.74–1.97)	0.45	1.07 (0.65–1.75)	0.79
**Donor**s						
VNRD	25594	1564 (6.11)	**Referent**			
FRD	44268	3519 (7.95)	1.33 (1.25–1.41)	**<.001**	0.88 (0.839–0.94)	**<0.001**
**Occupations**						
Healthcare workers	981	67 (6.83)	1.15 (0.89–1.49)	0.29	1.28 (0.98–1.66)	0.07
Students	19137	1327 (6.93)	1.17 (1.07–1.28)	**0.001**	1.33 (1.21–1.46)	**<.001**
Teachers/Researchers	1996	134 (6.71)	1.13 (0.93–1.37)	0.21	1.15 (0.95–1.407)	0.16
Police and armed forces	12362	741 (5.99)	**Referent**			
Drivers	3753	322 (8.58)	1.47 (1.28–1.69)	**<.001**	1.28 (1.11–1.47)	<0.001
Goverment employees	2426	155 (6.39)	1.07 (0.90–1.28)	0.46	1.01 (0.85–1.22)	0.88
Tailors and Hairdressers	5832	518 (8.88)	1.53 (1.36–1.72)	**<.001**	1.47 (1.30–1.66)	**<.001**
Hotel and restauration workers	740	68 (9.19)	1.59 (1.22–2.06)	**<0.001**	1.56 (1.20–2.03)	**<0.001**
Merchants	2934	246 (8.38)	1.44 (1.24–1.67)	**<.001**	1.40 (1.20–1.63)	**<.001**
Others	19701	1505 (7.64)	1.30 (1.18–1.42)	**<.001**	1.34 (1.22–1.45)	**<.001**

VNRD = Voluntary non-remunerated donors; FRD = Family/replacement donors; COR = Crude Odds Ratio; AOR = Adjusted Odds Ratio; CI = Confidence Interval;

^a^P-value for COR;

^b^P-value for AOR**.**

### Seroprevalence of HBsAg according to age, gender and donor type

Among females, there were no significant difference in HBsAg seroprevalence between FRD and VNRD except in the second largest group of 25–35 years (3.77% vs 5.03%, P = 0.01) (Table 4). Similarly, HBsAg seroprevalence was significantly higher in males FRD 18–45 years compared to VNRD (7.40% vs. 6.37%, P = 0.001; 8.40% vs 9.52%, P = 0.008 and 9.03% vs. 7.11%, P = 0.007) (Table 4).

**Table 4:** HBsAg seroprevalence according to age, sex and donor type.

Gender		Age groups									
		<18 years		18–24 years		25–35 years		36–45 years		>45 years	
		HBsAg+	%	HBsAg+	%	HBsAg+	%	HBsAg+	%	HBsAg+	%
**Female**	VNRD	4/136	2.94	161/4178	3.85	136/3603	3.77	50/815	6.13	12/225	5.33
	FRD	0/35	0.0	92/2299	4.00	177/3518	5.03	82/1281	6.40	21/382	5.50
	OR (95% CI)	NA		1.04 (0.79–1.36)		1.35 (1.07–1.71)		1.05 (0.72–1.53)		1.03 (0.47–2.28)	
	P-value	0.58		0.79		**0.01**		0.86		0.99	
**Male**	VNRD	8/106	7.55	474/7740	6.37	535/6369	8.40	143/2011	7.11	41/711	5.77
	FRD	7/136	5.15	890/12025	7.40	1633/17159	9.52	536/5937	9.03	81/1496	5.41
	OR (95% CI)	1.50 (0.48–2.04)		1.21 (1.08–1.30)		1.15 (1.03–1.27)		1.30 (1.07–1.58)		1.07 (0.71–1.60)	
	P-value	0.59		**0.006**		**0.008**		**0.007**		0.77	

VNRD = Voluntary non-remunerated donors; FRD = Family/replacement donors; OR = Odds Ratio; CI = Confidence Interval**.**

## Discussion

This study involved 69,862 first-time blood donors, nearly 82% of who were under 35 years old, reflecting the structure of the current young African population [16].

The overall seroprevalence of HBsAg was 7.28% in this study, confirming the WHO classification that places Gabon in a high-prevalence area (13). A previous study in Gabon in 2009 found a seroprevalence of HBV of 12.9% and 7.2% in urban and rural areas [10]. Compared to these data, the total seroprevalence (urban area) found in first-time donors in Libreville have dropped between 2009 and 2016. However, another recent report in Gabon showed a seroprevalence of 9.3% of HBV [17].

The seroprevalence in this study is much lower than the seroprevalences of 14.5%, 19.08% and 13.3% respectively found in the general populations of Burkina Faso, Togo and Ghana [18–20]. HBsAg seroprevalence has fluctuated annually, with a tendency to increase between 2009 and 2014 before decreasing from 2014 to 2016 to 6.5%. These variations in HBsAg seroprevalence cannot be explained by the tests performance from 2009 to 2016 as the NBTC used diagnostic immunoassays of the same sensitivity and specificity for HBsAg screening. The seroprevalence of HBsAg reported in the present study among first-time donor is lower than 12.6% showed in Cameroon; 8.89% in RCA; 10.01% in Equatorial Guinea, and 9.3% in Angola [5, 6, 21, 22]. However, it is comparatively higher than the seroprevalence of 4.1%, 3.21% and 2.58% obtained in Rwanda, Madagascar and Eritrea, respectively [23–25]. This could be explained by a difference in HBV epidemiology between the different geographical areas.

Men were almost twice more likely to be infected than women with HBV. These findings are consistent with several previous studies in Namibia, DRC, Burkina Faso, Mali, Turkey and Iran [26–31].

The seroprevalence of HBsAg was associated with the age of first-time blood donors with a more significant reactivity in donors aged 25–35 years. The age group of 25–35 years was 1.6 more times likely to be infected than the age group <18 years. The seroprevalence of HBsAg increased with the age of blood donors before decreasing after 45 years. Other earlier studies have shown an increase in HBV seroprevalence with age with peaks in the age groups 30–40 and 35–49 years in Morocco and Mexico [32, 33].

The profile of HBsAg seroprevalence according to age of first-time donors found in this study may be partially explained by HBV sexual transmission, re-exposure to the virus after loss of immunity, or chronic carriage of hepatitis B in blood donors infected prior to the introduction of the HBV vaccine in Gabon in 2004. The seroprevalence of HBsAg was associated with the type of donation in this study. FRD were significantly more infected compared to VNRD, corroborating WHO recommendations for the exclusive use of voluntary donations to obtain 100% blood donations from volunteers by 2020 [34]. However, FRD have been recommended in previous studies in sub-Saharan Africa [35, 36].

A recent study has shown that their use is critical and legitimate in low-income areas in developing countries to avoid blood shortages [37].

More specifically, the simultaneous comparison of HBsAg seroprevalence with blood donation type, gender and age showed a predominance of HBV infection among male FRD of the age groups 18–45 years and female FRD aged 25–35 years compared to VNRD, but this might be biased by differences in the populations in each age group, particularly regarding occupations. However, this is in agreement with the study by Maotela et al. which showed that volunteer donors <30 years old were at low risk of transfusion-transmissible infections [38]. Older family donors with low HBV infection may be a priority target population for blood donor recruitment.

The seroprevalence of HBsAg was associated with the socio-professional categories of blood donors. Hotel and Restaurant workers (9.19%), tailors and hairdressers (8.88%), drivers (8.58%), merchants (8.38%) and students (6.93%) were the most infected with HBV.

Overall, this study showed HBsAg seroprevalence of 6.83% among healthcare professionals. This is similar to the 7.00% seroprevalence reported in Tanzania [39]. Even HBV is a major threat to healthcare professionals [40], the risk of contracting this infection was similar that our reference population of police and armed forces. In contrast, an earlier study in Cameroon found that HBV infection was 4 times higher among healthcare workers [41]. The low seroprevalence of HBV in healthcare professionals could be due not only to a good knowledge of the transmission routes of this virus but also to better vaccination coverage of the latter.

This study is the first to give an estimate of the prevalence of HBsAg in a representative sample of the Gabonese urban adult population. It confirms the high endemicity of HBV in Gabon while identifying the most infected age groups for both men and women. It highlighted the need to provide vaccine and continuing education support on HBV mode of infection and transmission as well as other TTIs (HCV, HIV etc) to blood donors, particularly first-time blood donors.
